# Scrotal hemangioma: a case report and systematic literature review

**DOI:** 10.3389/fonc.2025.1586677

**Published:** 2025-08-20

**Authors:** Zhe Chang, Jiangwei Man, Jirong Wang, Huiming Gui, Jiping Niu, Li Yang

**Affiliations:** ^1^ Department of Urology, The Second Hospital of Lanzhou University, Lanzhou, China; ^2^ Gansu Province Clinical Research Center for Urology, Lanzhou, China; ^3^ Key Laboratory of Gansu Province for Urological Diseases, Lanzhou, China

**Keywords:** scrotum hemangioma, scrotum tumor, cavernous hemangioma, surgical treatment, diagnosis and prognosis

## Abstract

**Purpose:**

This study aimed to provide a case of scrotal hemangioma and examine its characteristics.

**Methods:**

We presented a case report involving a sixteen-year-old male, detailing symptoms, physical examination, imaging studies, diagnosis, and treatment. We conducted a thorough literature analysis of case reports and examined their clinical characteristics, pathological categorization, recurrence, and complications.

**Results:**

Our study comprised 21 patients with scrotal hemangioma. The average age was 27.14 years, with a range from 21 days to 84 years. 57.1% of the hemangiomas affected the organs. 61.9% of patients were situated on the right side of the scrotum. Ninety percent of patients experienced no pain. Merely 33.3% of patients exhibited additional symptoms, which encompassed azoospermia, hemorrhage, calcification, hydrocele, thrombosis, and ulceration. The predominant pathological classification was cavernous scrotal hemangioma, representing 38.1% of the cases. All patients received surgical resection, and the majority experienced neither relapse nor postoperative complications.

**Conclusions:**

Scrotal hemangioma, an uncommon benign neoplasm in adolescents, frequently remains asymptomatic but may disrupt fertility and, in rare instances, lead to severe problems. Timely identification, diagnosis, and surgical intervention are essential for good patient outcomes.

## Introduction

Hemangioma is a prevalent benign neoplasm of soft tissue resulting from vascular malformation, predominantly congenital, with a threefold higher frequency in girls compared to males (1). Hemangioma predominantly manifests in the head, neck, trunk, and limbs, along with the bladder and prostate, whereas the scrotum represents fewer than 1% of cases (2). Scrotal hemangioma can be classified into capillary, cavernous, racemose, and mixed types based on its clinical and pathological features (3). In addition to this, venous and anastomosing kinds of hemangioma also exist. The cavernous subtype of scrotal hemangiomas is the most commonly observed (4). The lesion generally manifests as thicker skin with soft lumps, along with dilated veins that resemble worms. It is bluish-purple or grayish-red, predominantly situated on the scrotum, and may impact adjacent tissues like the testicle, spermatic cord, and penis. The majority of patients with scrotal hemangioma report no pain; nevertheless, symptoms such as bleeding, ulceration, heaviness, or edema of the scrotum may occasionally manifest (5). The distinction among hemangioma, varicocele, and inguinal hernia primarily depends on imaging studies (6). Magnetic resonance imaging (MRI) is superior to ultrasound in diagnosing and differentiating scrotal hemangioma. Like vascular tumors in other regions, scrotal hemangiomas are often managed with surgical excision. Moreover, other therapeutic modalities encompass laser therapy, intervention, and sclerotherapy (7–9). This condition seldom recurs post-surgery, with little postoperative consequences. The essential factor in its therapy is the precise preoperative diagnosis and a comprehensive understanding of its anatomical interaction with adjacent tissues.

## Methods

### Search strategy

**Figure 1:**
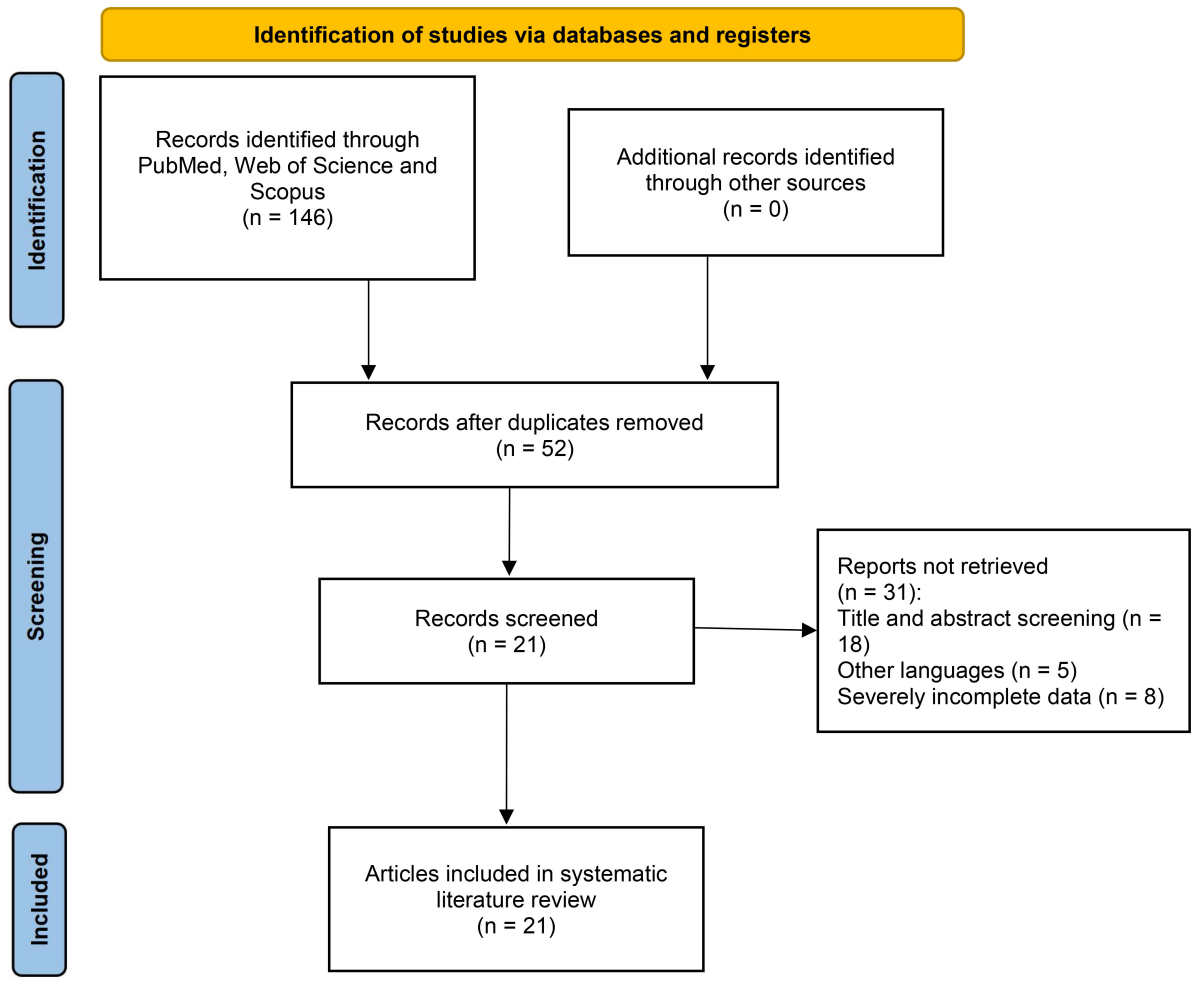
A PRISMA flow diagram illustrating the screening of cases.

This systematic literature review was conducted independently by two reviewers following the Preferred Reporting Items for Systematic Reviews and Meta-Analyses (PRISMA) standards (CRD420251116989) to enhance understanding of the subject and provide valuable insights to the medical industry. Our investigation encompassed querying the PubMed, Web of Science, and Scopus databases. The search utilized a systematic amalgamation of phrases focusing on both the scrotum and angioma, specifically: [(Scrotum OR Scrotal) AND (Hemangioma OR Angioma OR Hemangiomas)]. The search occurred on 30 January 2025. Furthermore, the references were verified to prevent omissions (**Figure 1**).

### Inclusion and exclusion criteria

All studies in the English language concerning scrotal hemangioma were incorporated. The hemangioma was situated in the scrotum, rather than coming from other organs, and may have involved more organs. Studies and reviews in non-English languages were removed from our research. Due to the limited number of accessible instances, we classify patients with incomplete clinical characteristics as uncertain.

### Case selection, data extraction, and quality assessment

Two researchers independently assessed paper titles and abstracts utilizing established search parameters to identify studies that fulfilled the inclusion criteria. The two identical researchers evaluated the entire texts for inclusion and collected data. Subsequently, all selected cases were analyzed, and the information was gathered using Excel.

Clinical characteristics were gathered for each included case, encompassing age, tumor location, and its relation to adjacent organs, pain and other symptoms, pathological categorization, recurrence, and complications. The baseline analysis was performed on the gathered instances using R (http://www.Rproject.org).

## Results

### Case report

A sixteen-year-old Chinese male had a walnut-sized purple mass on the left side of his scrotum, measuring approximately 4cm by 4cm. The first lesion was discovered a week ago, and since then, its size has progressively increased, accompanied by discomfort upon palpation, without any swelling. The patient reported no history of trauma, abdominal or pelvic surgery, congenital disorders, or familial hereditary problems.

**Figure 2:**
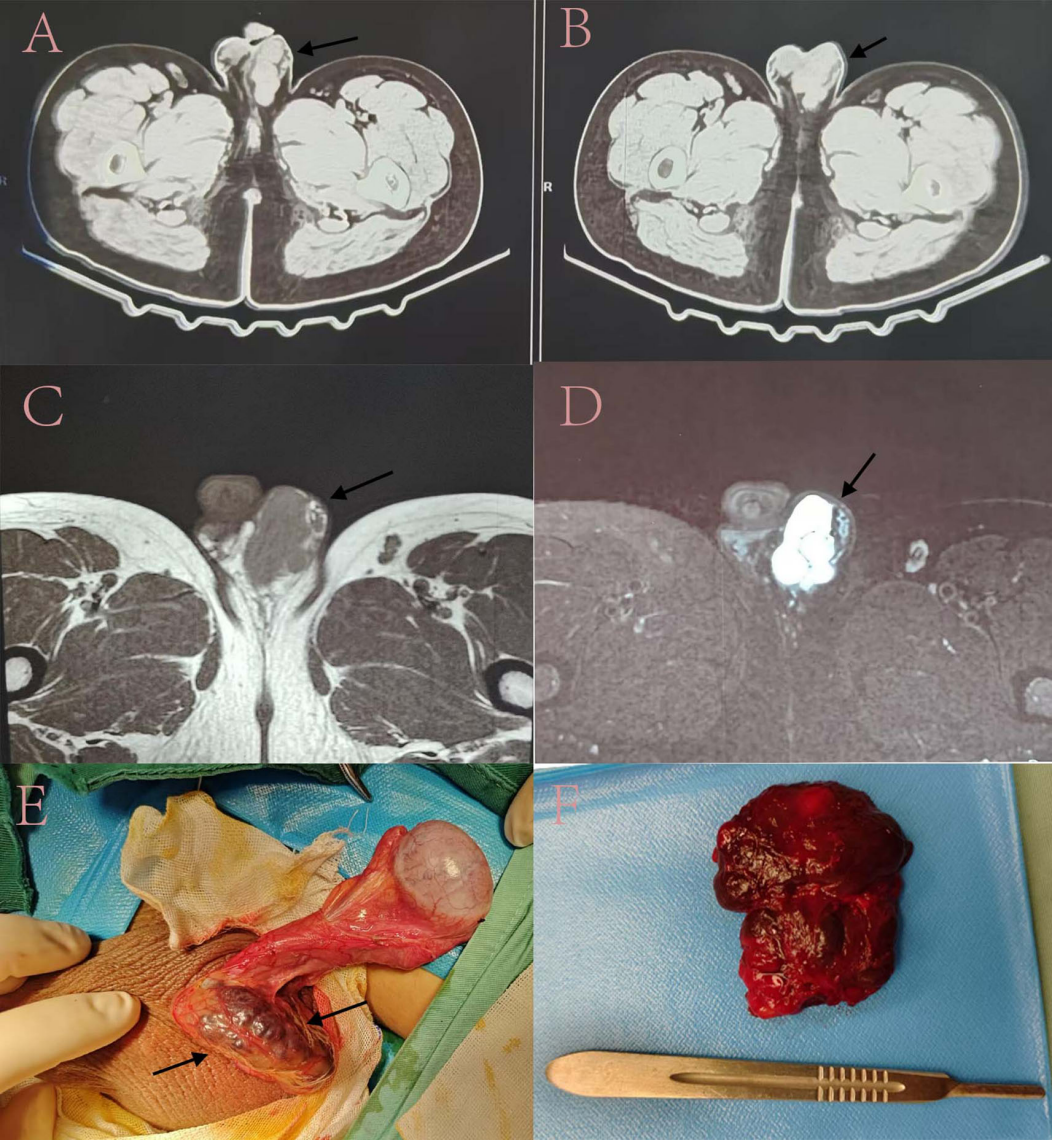
Imaging assessment and intraoperative observations. **(A, B)**. The results of Pelvic Computed Tomography; **(C, D)**. The results of magnetic resonance imaging; **(E, F)**. The tumor’s position during the surgical procedure and the subsequent specimen obtained postoperatively. Numerous black arrows denote the tumor’ s position.

Ultrasound imaging revealed a hypo-echoic, irregularly shaped mass in the left epididymis head region, measuring 5.5 cm × 4.7 cm × 3.2 cm, characterized by distinct borders and internal hyperechoic septations, with no evident blood flow signals. A Computed Tomography (CT) scan of the pelvis indicated a lobulated soft tissue mass on the left testis, accompanied by several enlarged inguinal lymph nodes bilaterally (**Figures 2A, B**). The MRI demonstrated a consistently isointense T1 and hyperintense T2 signal mass with many septations in the left testicular tunica albuginea, exhibiting a strong signal on diffusion-weighted imaging (DWI) (**Figures 2C, D**). All laboratory tests were routine, particularly Serum Alpha-Fetoprotein (1.26 ng/ml) and Human Chorionic Gonadotropin (0.01 Miu/ml).

**Figure 3:**
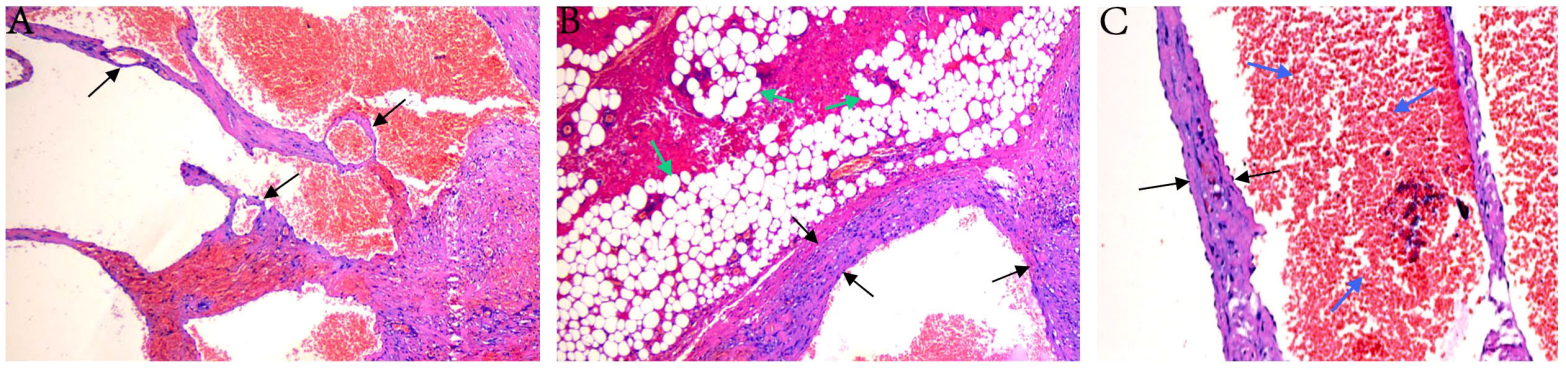
Histopathological images. **(A)** Large, dilated vascular channels containing red blood cells (RBCs) within their lumina. **(B)** Dilated, thick-walled blood vessels surrounded by mature adipose tissue. **(C)** High-power view showing dilated blood vessels with intraluminal RBCs and prominent endothelial lining. The black arrows indicate vessel walls, green arrows indicate adipocytes, and blue arrows indicate RBCs.

We conducted a surgical excision for the patient. A clear demarcation was noted between the hemangioma and the testis during the surgical investigation. Therefore, the choice was made to retain the testis while excising solely the hemangioma (**Figures 2E, F**). The postoperative pathology report indicated a cavernous hemangioma. The tumor displays a lobulated architecture consisting of several proliferating tiny blood vessels with thin walls (**Figure 3**). Post-operation, the patient exhibited no discernible discomfort at the surgical site and had satisfactory recovery. Telephone follow-up at nine months post-surgery revealed the patient experienced no postoperative problems or recurrence.

### Result of baseline analysis

Our investigation comprised a total of 21 patients with scrotal hemangioma (2, 5, 10–28). The age varied from 21 days to 84 years, with a mean age of 27.14 years. In 57.1% of instances, the hemangioma affected genital structures, including the penis, spermatic cord, or testicle. Sixty-one point nine percent of patients were situated on the right side of the scrotum. Ninety percent of patients experienced no pain. 33.3% of patients exhibited additional symptoms or complications, including azoospermia, hemorrhage, calcification, hydrocele, thrombosis, and ulceration. The predominant pathological classification was cavernous scrotal hemangioma, comprising 38.1% of the cases, followed by capillary hemangioma at 19.1%. Despite the application of alternative therapeutic modalities, all patients underwent surgical resection. The majority of patients experienced neither recurrence nor surgical complications (**Table 1**).

**Table 1:** The baseline analysis of the 21 cases with scrotal hemangioma.

Clinical features	Level	Overall
N		21 (%)
Age [mean (SD)[		27.14 (24.07)
With organ (%)	No	9 (42.9)
	Penis	2 (9.5)
	Spermatic cord	3 (14.3)
	Testicle	7 (33.3)
Location (%)	Left	5 (23.8)
	Middle	1 (4.8)
	Right	13 (61.9)
	Unknown	2 (9.5)
Pain (%)	No	19 (90.5)
	Yes	2 (9.5)
Other symptoms (%)	No	14 (66.7)
	Bleeding	2 (9.5)
	Calcifications	1 (4.8)
	Hydrocele	1 (4.8)
	Azoospermia	1 (4.8)
	Thrombosis	1 (4.8)
	Ulcer	1 (4.8)
Pathological classification(%)	Cavernous	8 (38.1)
	Capillary	4 (19.1)
	Venous	2 (9.5)
	Mixed-type	2 (9.5)
	Racemose	1 (4.8)
	Anastomosing	1 (4.8)
	Unknown	3 (14.3)
Relapse (%)	No	13 (61.9)
	Penis	1 (4.8)
	Unknown	7 (33.3)
Complication (%)	No	14 (66.7)
	Unknown	7 (33.3)

## Discussion

Hemangioma, specifically cavernous hemangioma, is an uncommon congenital condition in the scrotum. Scrotal hemangiomas predominantly manifest in youngsters, with 70%-90% of instances occurring before the age of 7 (29). Nevertheless, a limited number of patients with the illness display significant symptoms or consequences, especially when the lesion is diminutive and progresses gradually. Attracting patients’ early attention is challenging unless a mass is identified during inspection. The individuals identified and treated are, in fact, adolescents or even adults. The lesion manifests as a pliable mass under the scrotal skin or as many engorged veins creating a serpentine mass with a bluish-purple or gray hue. It is frequently noted in the scrotal wall or within the scrotum. This illness may be readily misinterpreted as varicocele, lymphangioma, or arteriovenous malformation (27, 30). Hemangiomas generally present unilaterally and, when substantial in size, may involve the spermatic cord, testis, penis, and spread to the perineum and buttocks (28). It is essential to differentiate scrotal hemangioma from benign and malignant malignancies of these organs.

Patients may exhibit varying tumor-related abnormalities or other symptoms based on the location, size, and level of hemangioma involvement. A deep scrotal hemangioma exhibiting ulceration, pruritus, and hemorrhage was documented (31), and thrombosis was identified within the scrotal hemangioma (12). Some patients may present with additional findings such as calcifications or associated hydroceles (18, 21). A patient with scrotal hemangioma had azoospermia resulting from testicular injury induced by elevated temperatures associated with the condition (5).

Imaging examination, as the primary diagnostic instrument, is essential for enabling doctors to diagnose hemangiomas accurately. This method not only aids in the detection of hemangiomas but also assists in excluding alternative differential diagnoses. Ultrasound is a fundamental assessment that can initially ascertain the characteristics and dimensions of scrotal malignancies. Manifestations encompass robust blood flow signs and a variable arterial spectrum (26). Nevertheless, because of the ambiguous presentation of the tumors, the precision of ultrasound is limited, and at times, it is challenging to differentiate them from varicoceles. CT is essential for assessing hemangiomas with calcification or venous calculi. MRI imaging provides exceptional clarity for malignancies, especially soft tissue tumors, facilitating the identification of anatomical relationships between tumors and adjacent tissues to determine suitable surgical approaches (6). Pathological examination is unequivocally the gold standard for diagnosis.

Surgical excision is generally selected for the management of scrotal hemangioma. When the tumor infiltrates the internal organs of the scrotum, the surgical approach is determined by the extent of this infiltration. Some people have undergone orchiectomy, and although the contralateral testicle is preserved, it still affects the patient. Consequently, partial testicular resection may be deemed appropriate. In addition, there are also treatments including arteriosclerosis (8, 9), laser (32, 33)and medication such as propranolol (34). In young patients, these methods carry significant risks of recurrence and consequences, and their application is not prevalent. A patient with a congenital hemangioma on the penis and scrotum underwent interventional embolization and achieved a favorable prognosis. In contrast to malignant tumors, scrotal hemangiomas, being benign, exhibit little invasive potential and recurrence, with the majority of patients experiencing a favorable prognosis.

## Conclusions

This study presents a case report of a 16-year-old boy with scrotal hemangioma, outlining the diagnostic procedure and treatment management. Furthermore, we executed an exhaustive examination of 21 previously documented instances from the literature and analyzed their demographic and clinical attributes. These findings offer critical insights to aid clinicians in the early diagnosis and appropriate treatment of this illness.

## Data Availability

The original contributions presented in the study are included in the article/Supplementary Material. Further inquiries can be directed to the corresponding author.
